# Blood and saliva‐derived ctDNA is a marker of residual disease after treatment and correlates with recurrence in human papillomavirus‐associated head and neck cancer

**DOI:** 10.1002/cam4.6191

**Published:** 2023-08-01

**Authors:** Sarah Tadhg Ferrier, Thupten Tsering, Nader Sadeghi, Anthony Zeitouni, Julia V. Burnier

**Affiliations:** ^1^ Cancer Research Program Research Institute of the McGill University Health Centre Montreal Canada; ^2^ Department of Pathology McGill University Montreal Canada; ^3^ Department of Otolaryngology – Head and Neck Surgery McGill University Montreal Canada; ^4^ Gerald Bronfman Department of Oncology McGill University Montreal Canada

**Keywords:** biomarker, circulating tumor DNA, head and neck cancer, human papillomavirus, liquid biopsy, minimal residual disease, treatment response

## Abstract

**Background:**

There is an alarming increase in human papillomavirus‐associated head and neck cancer (HNC), reaching epidemic levels. While patient prognosis is generally good, off‐target treatment effects are associated with decreased quality of life. Thus, non‐invasive strategies to predict treatment response and risk of recurrence could help de‐escalate treatment. In this study, we tested circulating tumor (ct)DNA in liquid biopsies (blood/saliva) of HPV‐positive HNC patients to assess treatment response and disease progression.

**Methods:**

A total of 235 blood and saliva samples were collected from 60 HPV‐positive and 17 HPV‐negative HNC patients (control group) before and/or after treatment. Samples were analyzed using ddPCR for HPV16/18/31/33/35/45 and correlated with imaging and pathological examination.

**Results:**

HPV‐ctDNA detection was significantly higher prior to treatment (91%) than after treatment (8.0%) (*χ* ^2^ *p* < 0.00001), with high concordance between saliva and blood (93%). In matched samples, all patients positive for ctDNA before treatment showed significant reductions in ctDNA levels post treatment (*p* < 0.0001). All but one patient with persistent ctDNA after treatment showed residual tumor and subsequent recurrence. Finally, fragmentomic analysis revealed shifts in cell‐free DNA fragment size after treatment, suggesting a complementary biomarker for treatment response.

**Conclusions:**

Blood and saliva were found to be good sources of HPV‐ctDNA. The presence of ctDNA strongly correlated with treatment response, demonstrating clinical utility as a non‐invasive biomarker to monitor tumor progression in HPV‐positive HNC. Liquid biopsy based ctDNA testing could be an effective approach to predict recurrence and stratify patients for de‐escalation of treatment, thereby improving quality of life.

## 1 BACKGROUND

Head and neck cancer (HNC) is the sixth most common cancer worldwide, accounting for 890,000 cases and 450,000 deaths annually.^1^, ^2^ Current treatment regimens are associated with significant side effects such as difficulty swallowing and speaking.^3^ In recent decades, incidence rates of certain subtypes of HNC—including oral cavity squamous cell carcinomas (SCCs)—have decreased due to changes in smoking and other lifestyle factors.^4^ Despite this, the prevalence of oropharyngeal SCC has increased alarmingly, related to human papillomavirus (HPV) infection rates, with these cases generally occurring in a younger patient population.^1^, ^4^, ^5^ In the US and part of the EU, an estimated 60%–70% of newly diagnosed oropharyngeal cancers (OPCs) are related to HPV infection.^6^ Treatment for HPV‐related HNC includes radiation or surgery, with or without systemic therapy.^6^ HPV‐related OPC patients show significantly longer survival than HPV‐negative patients, with a median survival of 131 versus 20 months.^7^ Because of the favorable prognosis, the goal of many ongoing trials is to de‐escalate treatment to reduce treatment‐related toxicity without compromising efficacy.^8^

Multiple oncogenic HPV strains can be responsible for the development of OPC. The most prevalent strain is HPV16, accounting for 90% of cases of HPV‐related tumors,^5^ followed by HPV18 and HPV33.^9^ Tumors are generally diagnosed as HPV+ through surrogate immunohistochemical staining for p16, but p16 staining and HPV status are not always concordant.^10^

While HPV has been causally linked to the development of HNC for decades,^5^ there are currently no standardized options for HPV screening, analogous to the pap test for cervical cancer.^11^, ^12^ Liquid biopsy involves the analysis of bodily fluids such as blood, saliva, and urine. Cell‐free (cf)DNA are short fragments of DNA that are released from cells as a result of cell death—including apoptosis and necrosis—and through active secretion.^13^ Circulating tumor (ct)DNA refers to the cfDNA that is specifically released from tumors and can be identified using features of the cancer cell of origin, including mutations and viral sequences. ctDNA has been used as a biomarker to monitor tumor status and correlate with treatment response in many solid malignancies.^11^ With a very short half‐life in circulation (minutes to hours), ctDNA gives a real‐time assessment of disease at the time of sample collection.^14^ While most ctDNA studies have focused on genomic events, viral ctDNA has been detected in patient blood for numerous virus‐related tumor types, including cervical cancer,^15^ Burkitt's lymphoma,^16^ and nasopharyngeal^17^ and oropharyngeal carcinomas.^18^ In these tumors, viral ctDNA is associated with disease presence and progression.

Due to the side effects caused by treatment of advanced OPC, as well as the potential for late recurrences in these patients, a non‐invasive method to monitor treatment response and disease progression would be ideal, preventing overtreatment and the associated long‐term side effects. In this prospective study, we analyzed 235 saliva and blood samples to determine the clinical utility of ctDNA as a biomarker of treatment response and to predict recurrence.

## 2 METHODS

### 2.1 Patient samples

Following informed consent, 77 patients were enrolled in this study at the Royal Victoria Hospital Head and Neck Clinic in accordance with ethics protocol 2020‐5918 approved by the Research Institute – McGill University Health Centre Review Ethics Board. Of these patients, 60 had confirmed p16+ tumors, while 17 were being treated for HPV‐unrelated (p16‐) HNC. Blood and saliva samples were obtained from patients. Patient history, including age, sex, prior scans (PET, MRI, CT) and results from pathological and physical examination were used to correlate ctDNA levels with clinical history. Patients were staged according to American Joint Committee on Cancer 8th edition (AJCC‐8). Patients were classified based on treatment, sampling time (pre‐treatment, post‐chemotherapy/post‐radiation/post‐surgery), and whether they were currently in treatment for a newly diagnosed tumor or recurrence. Patients who did not have follow‐up imaging or pathology were excluded from analysis.

### 2.2 Blood and saliva collection and sample processing

10 mL blood samples were collected from patients from a peripheral vein or central line in PAXgene blood ccfDNA tubes (768165, BD Biosciences). Samples were centrifuged at 2000*g* for 15 min to isolate plasma, followed by a second spin at 2000*g* for 10 min to remove any remaining cells. Buffy coat was isolated after the first round of centrifugation.

2 mL saliva samples were collected in Oragene self‐collection tubes (OG‐600, DNA genotek) by patients according to the MD Anderson protocol, as previously described by Wang et al.^19^ In brief, patients were asked to allow saliva to collect in the floor of the mouth for 5 min before spitting into a collection vial. Upon filling the vial, the self‐collection tubes were closed to release the DNA preservation solution, and were inverted 12 times. Samples were centrifuged at 2000*g* for 15 min to remove cellular matter from cell‐free saliva. Samples were then centrifuged at 2000*g* for 10 min. If cellular matter was present after the second centrifugation step, this step was repeated. Samples were stored at −80°C until DNA extraction.

### 2.3 DNA extraction from plasma and saliva

Cell‐free DNA was extracted with the Circulating Nucleic Acid Kit (55114, Qiagen) using the protocol for extraction of DNA from 4 mL serum or plasma, as routinely performed in our lab, modified through the use of a two‐step elution. For plasma samples, elution was performed first with 20 μL followed by 10 μL buffer AVE. For saliva samples, elution was performed with 40 μL and then 20 μL buffer AVE. For each elution step, buffer was left in the tubes for 5 min and then spun for 2 min at 20000*g*.

DNA was quantified using the Qubit fluorometer dsDNA high sensitivity kit (Q32854, Invitrogen) following the manufacturer's protocol. Samples were briefly vortexed and centrifuged before quantification using 1 μL of DNA.

DNA fragment size was determined using the Agilent 2100 Bioanalyzer with the High Sensitivity DNA kit according to the manufacturer's protocol. Dominant fragment size was determined as the bioanalyzer peak with the higher molar concentration in the sample.

### 2.4 PCR conditions

For all patients, ddPCR was performed first using primers/probes for HPV16 E7 and RPP30 (reference gene). If negative results were obtained for HPV16, the reaction was performed with primers/probes for HPV18 E7 and HPV33 E7. ddPCR was performed according to the manufacturer's protocol, using 10 μL 2× ddPCR Supermix for probes, 900 nm primers (Integrated DNA Technologies), 250 nm probes (FAM/HEX, Affinity Plus qPCR Probes, Integrated DNA Technologies), up to 8 μL of DNA template, and nuclease‐free water. For patients negative for HPV16/18/33, samples were tested using ddPCR with EVAgreen and primers for HPV31/35/45. For each reaction, the mix described above was added to the cartridge, followed by 70 μL of Droplet Generation Oil (1863005, Bio‐Rad Laboratories). Droplets were generated using the QX200 Droplet Generator (Bio‐Rad Laboratories). PCR was performed as follows: 1 × 95°C (10 min), 45 × (95°C (30 s), 60°C (60 s), and 72°C (30 s), and 1 × 90°C (10 min). The plate was read using the QX200 Droplet Reader (Bio‐Rad Laboratories), and data was analyzed with QuantaSoft software. All samples were performed in duplicate. Samples with <10,000 droplets were excluded from analysis.

### 2.5 Primer and probe sequences

HPV16 E7 F: TGTGACTCTACGCTTCGGTTG

HPV16 E7 R: GCCCATTAACAGGTCTTCCA

HPV16 E7 probe: /56‐FAM/TA + CAAA+GCACA+CA + CGT/3IABkFQ/

HPV18 E7 F: GCATGGACCTAAGGCAACAT

HPV18 E7 R: GAAGGTCAACCGGAATTTCAT

HPV18 E7 probe: /5HEX/CA + TT + GTATT+G + CATTTA+GA + GCC/3IABkFQ/

HPV31 E7 F: TGCGTGGAGAAACACCTACG

HPV31 E7 R: AACAGTGGAGGTCAGTTGCC

HPV45 E7 F: GGCAACACTGCAAGAAATTGTA

HPV45 E7 R: CCTCCTCTGACTCGCTTAATTG

HPV 33 E7 F: CTTGTAACACCACAGTTCGTTTATG

HPV 33 E7 R: GTGCCCATAAGTAGTTGCTGTA

HPV33 E7 probe: /56‐FAM/AAGT+G + A + CC + T + A + CGA/3IABkFQ/

HPV35 E7 F: TGGAGAAATAACTACATTGCAAGAC

HPV35 E7 R: GCTGTCACACAATTGCTCATAA

RPP30 F: AGCCCTAATGTTCACAGCTC

RPP30 R: TTGCTTTGTGGCCTAGGTATTA

RPP30 probe: /5HEX/TC + C + T + CTCT+GC + CTA/3IABkFQ/

### 2.6 Statistical analysis

Assay performance was assessed using all patient samples including control patients. Disease presence was determined on the basis of imaging and/or pathology results. Patients were considered to have present disease when they showed signs of disease either at the time of sampling or a time point following sample collection without intervening treatment. Patients were considered to be negative for disease if they had confirmed HPV‐negative HNC or if they had successful treatment with no signs of recurrence or residual disease in at least the 6 months following sample collection. Patients with inconclusive pathological and imaging results were excluded from testing. Sensitivity was calculated as true positive samples/total samples where patients had active disease. Specificity was calculated as true negative samples/total disease negative samples.

## 3 RESULTS

### 3.1 High concordance in ctDNA detection was seen between blood and saliva liquid biopsies

Blood and saliva samples were collected from 60 HPV‐positive and 16 HPV‐negative HNC patients (control group) before and/or after surgical resection. Patients with confirmed p16‐positive primary or recurrent oropharyngeal SCCs at any stage of treatment were included. For the control group, patients with oral cancers unrelated to HPV were included. All patients had confirmed follow‐up scans and/or pathology. Patient characteristics are shown in Table 1. Of the 60 patients, the median age was 66 years, with a predominance of men (82%). The primary tumor was located in the tonsil in 36 patients and the base of tongue in 24 patients. Tumor stages ranged from T1N1 to T4N2. Primary treatments were induction chemotherapy using cisplatin and docetaxel followed by surgery in 70% of patients (NECTORS trial, NCT04277858), and chemoradiation in 23% of patients. Remaining patients received treatments including surgery alone and surgery combined with chemoradiation. Patients in treatment for recurrence (*n* = 5) received either surgery or chemoradiation.

**TABLE 1:** Patient characteristics.

	HPV+ HNC	HPV− HNC (control)
Total patients	60	17
Sex (M/F)	49/11	15/2
Median age (range)	66 (42–84)	
Primary/recurrent	55/5^a^	N/A
Plasma available	55/60 patients	17/17 patients
Saliva available	57/60 patients	14/17 patients
Treatment	Chemotherapy and surgery: 42 patients Chemoradiation: 14 patients Other: 4 patients	N/A

^a^ One patient was additionally sampled for later recurrence following primary disease.

**FIGURE 1:**
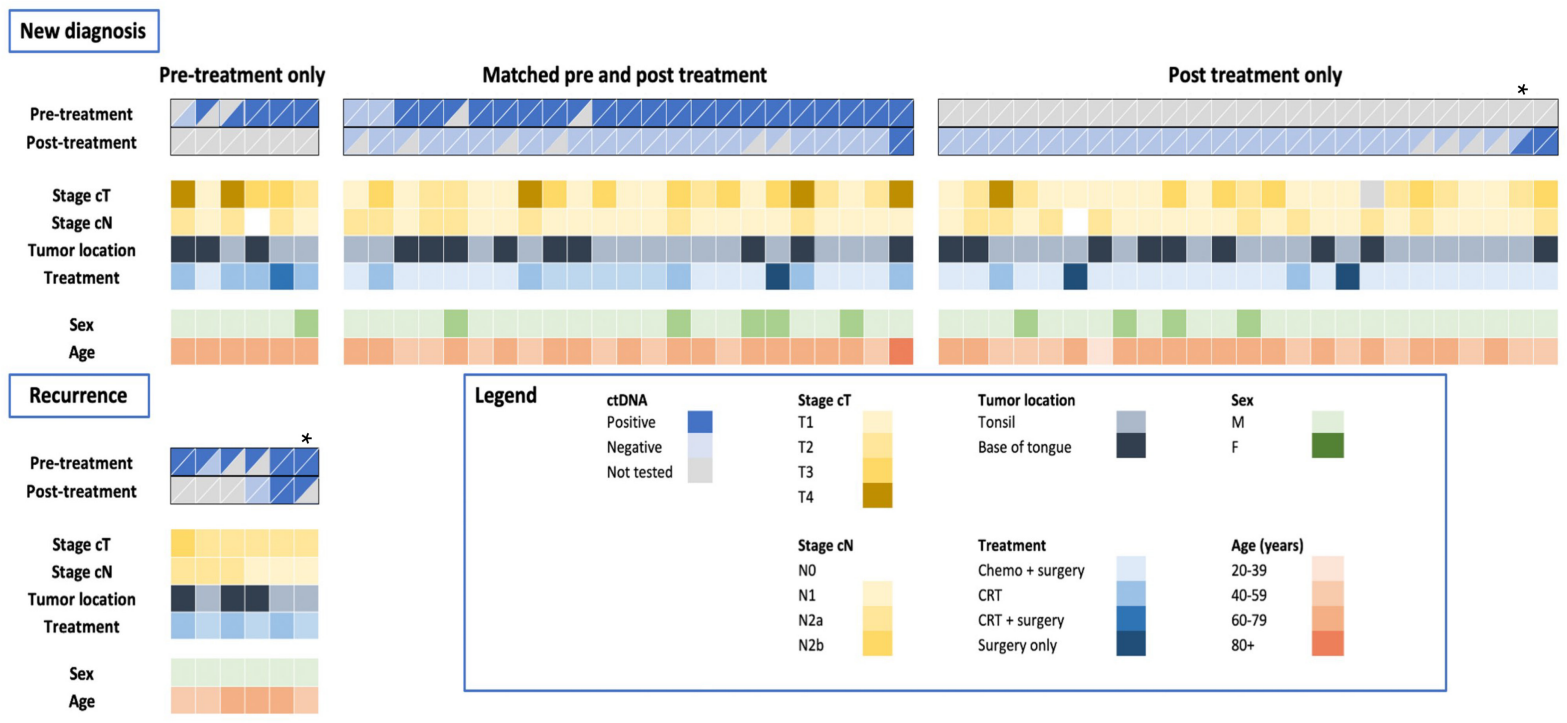
Patient characteristics and ctDNA results for all patients. Patient characteristics (sex and age) and tumor characteristics (new or recurrent, stage and location) are shown. Plasma and saliva ctDNA results is indicated before and after treatment, separated for patients with only pre‐treatment samples, patients with matched pre‐ and post‐treatment samples, and patients with only post‐treatment samples. Gray shading indicates not available data. *One patient was sampled for both primary disease and later at recurrence.

**FIGURE 2:**
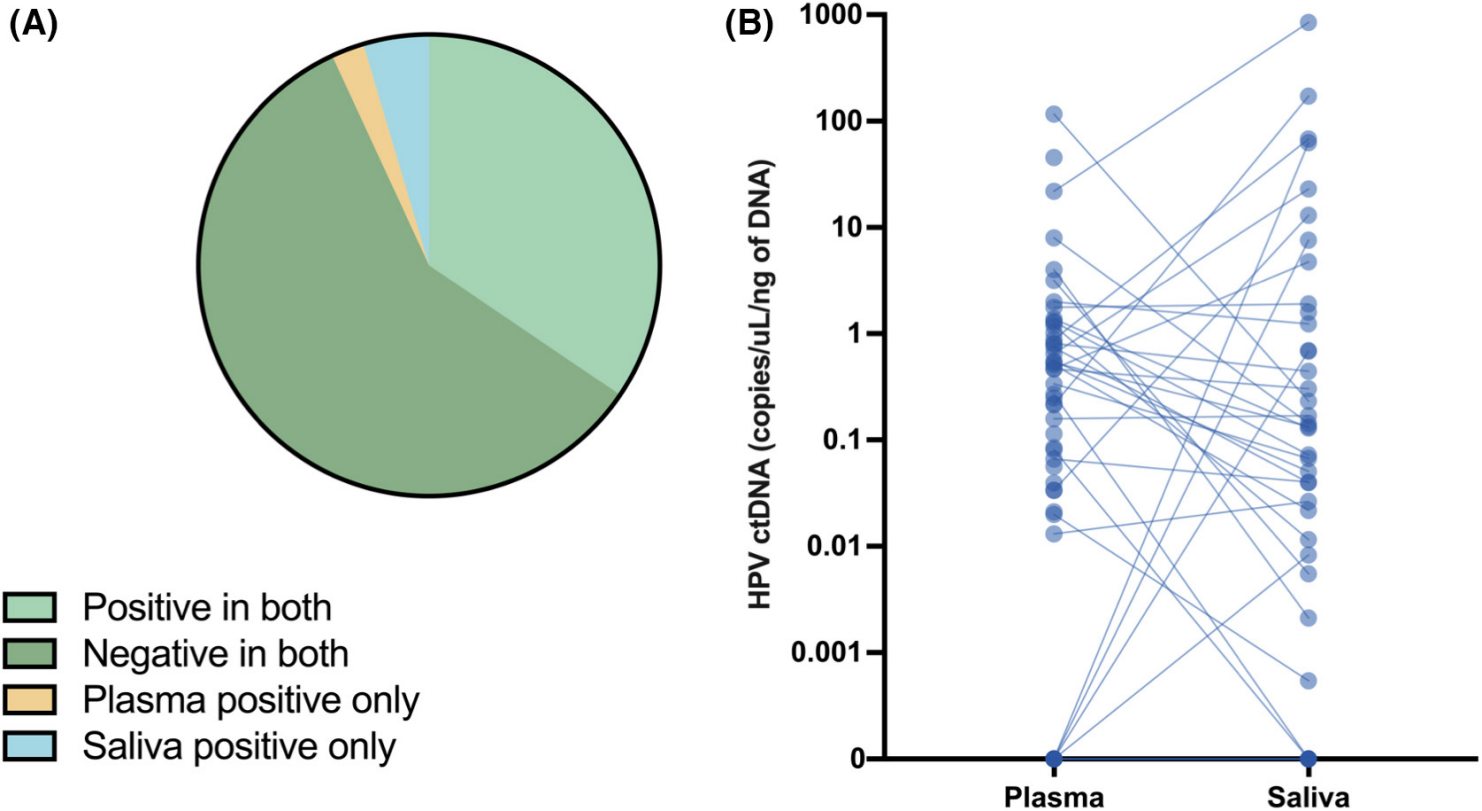
ctDNA presence in saliva and plasma shows a high degree of concordance. (A) Overall concordance between plasma and saliva ctDNA results across all matched samples. (B) Plasma and saliva HPV‐ctDNA (copies of HPV/μL/ng of DNA) levels across all samples.

All plasma and saliva samples were tested for HPV‐ctDNA using ddPCR (Figure 1). As expected, all control patients with confirmed p16‐negative oral tumors (*n* = 17) showed no detectable HPV‐ctDNA in either plasma or saliva. A high rate of concordance in ctDNA positivity was found between blood and saliva (93%, 81/87 matched blood and saliva samples, Cohen's kappa = 0.821, Figure 2A,B). Testing both blood and saliva increased the sensitivity of the assay in patients who were only positive in one analyte, as certain patients with active disease were not positive in both blood and saliva, potentially due to differences in plasma cfDNA levels as well as capability to produce saliva.

### 3.2 ctDNA detection in both saliva and blood was significantly higher before treatment compared to after treatment

**FIGURE 3:**
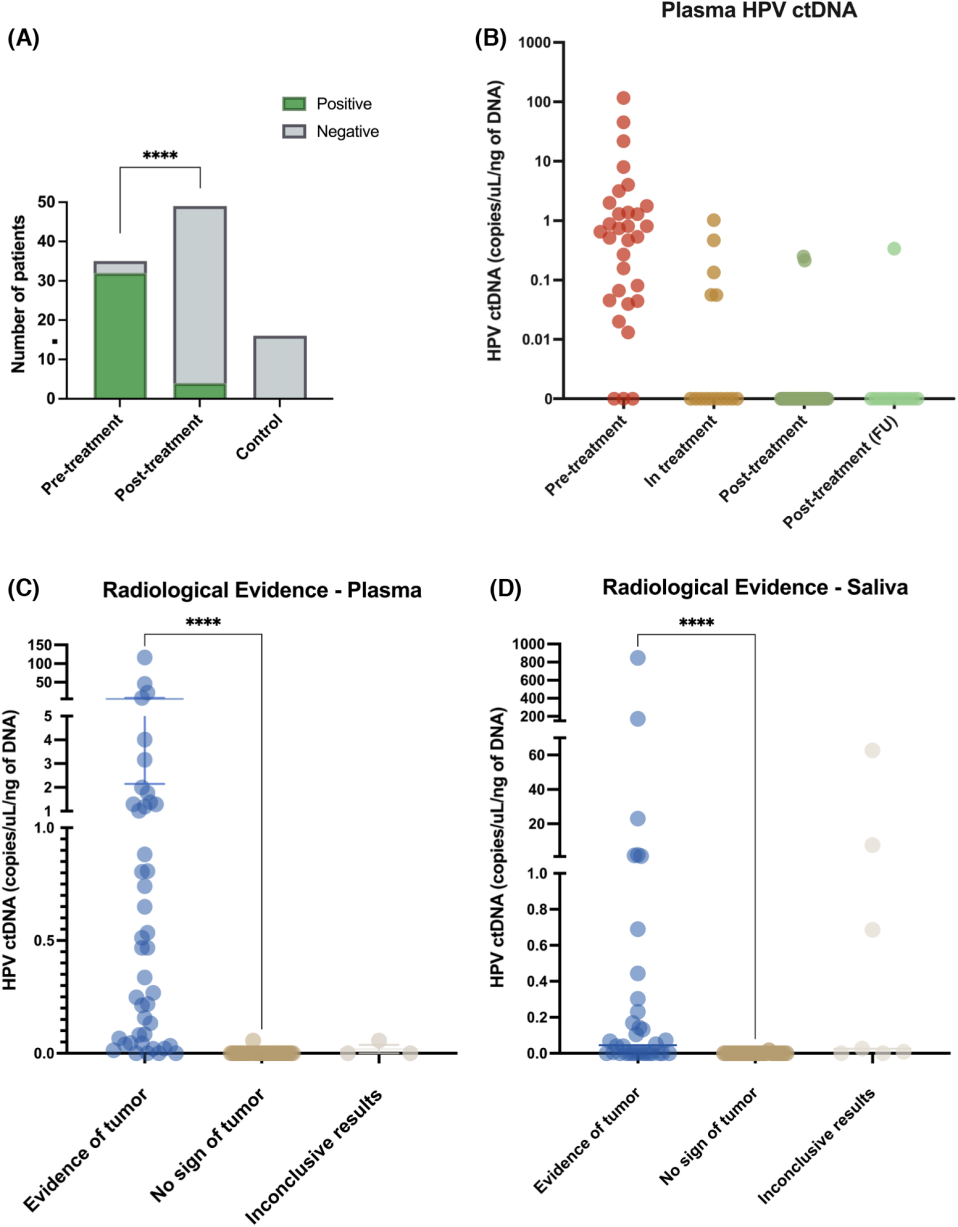
ctDNA levels are significantly higher before treatment and correlate to treatment response. (A) ctDNA detection by PCR for HPV16/18/33 sequences shown for pre‐ and post‐treatment samples, *χ* ^2^ *p* < 0.0001. (B) ctDNA levels (copies/μL/ng of DNA) shown according to treatment stage. Levels of HPV‐ctDNA (copies/μL/ng of cfDNA) categorized by evidence of tumor in either pathological analysis or imaging for ≥6 months post sample collection for (C) plasma and (D) saliva., Mann Whitney *p* < 0.0001 for patients with and without radiological evidence of tumors.

ctDNA has been shown to be an important biomarker of treatment response in many solid malignancies.^20^, ^21^ Here, we sought to determine whether the presence of ctDNA correlated with treatment response. In our HPV+ HNC cohort, ctDNA was detectable in 32/35 (91%) patients prior to treatment and 4/50 (8.0%) post‐treatment (*χ* ^2^ *p* < 0.00001, Figure 3A). When analyzed separately, both blood and saliva showed significant differences in ctDNA detection between pre‐treatment (blood: 31/33 [94%]; saliva: 27/30 [90%]) and post‐treatment sampling (blood: 3/53 [5.7%]; saliva: 4/41 [9.8%]) as shown in Figure 3B and Table 2 (*χ* ^2^ *p* < 0.00001).

**TABLE 2:** ctDNA by treatment category.

	Positive in blood & saliva	Positive in blood or saliva	Negative	Total patients
Pre‐treatment patients	24^a^	32	3	35
Post‐treatment patients	4	4	46	50
Control patients (HPV− HNC)	0	0	17	17

^a^ Saliva unavailable for 5 patients, blood unavailable for 2 patients.

For the 3 p16+ patients who were negative for ctDNA prior to treatment, we could not detect HPV16/18/31/33/35/45 DNA in the tumor tissue (when available) and/or liquid biopsy samples, suggesting that they may have been p16 positive without associated HPV infection, similar to previous reports.^9^, ^10^ Because the patients who showed undetectable pre‐treatment ctDNA had relatively large tumors, these data would suggest that we may have 100% detection of HPV‐ctDNA pre‐treatment in our cohort when accounting for HPV status, since a higher disease burden is generally correlated with increased ctDNA presence.

### 3.3 ctDNA detection was highly correlated with treatment response, residual disease and recurrence

As ctDNA detection was clearly higher in pre‐treatment samples, we next sought to determine whether ctDNA correlated with treatment response. All 46 patients (100%) who tested negative for ctDNA following treatment showed no signs of active disease in follow‐up scans and examination, with an average post‐treatment follow‐up of 818 days (range: 199–1581). In contrast, 3 of the 4 patients (75%) who had positive ctDNA post‐treatment demonstrated signs of residual tumor or recurrence. These patients remained ctDNA‐positive in follow up samples, demonstrating a clear relationship between treatment response and post‐treatment ctDNA. Indeed, when comparing results to imaging or pathology, the presence of HPV‐ctDNA was extremely highly associated with disease status (*p* < 0.0001, Figure 3B–D). Taken together, this data demonstrates that both blood and saliva are effective tools for monitoring disease status, including residual disease and recurrence. The assay demonstrated a sensitivity of 93.48%, specificity of 98.72%.

### 3.4 In matched longitudinal samples, ctDNA decreased significantly after treatment, with positive ctDNA after treatment indicating residual disease

**FIGURE 4:**
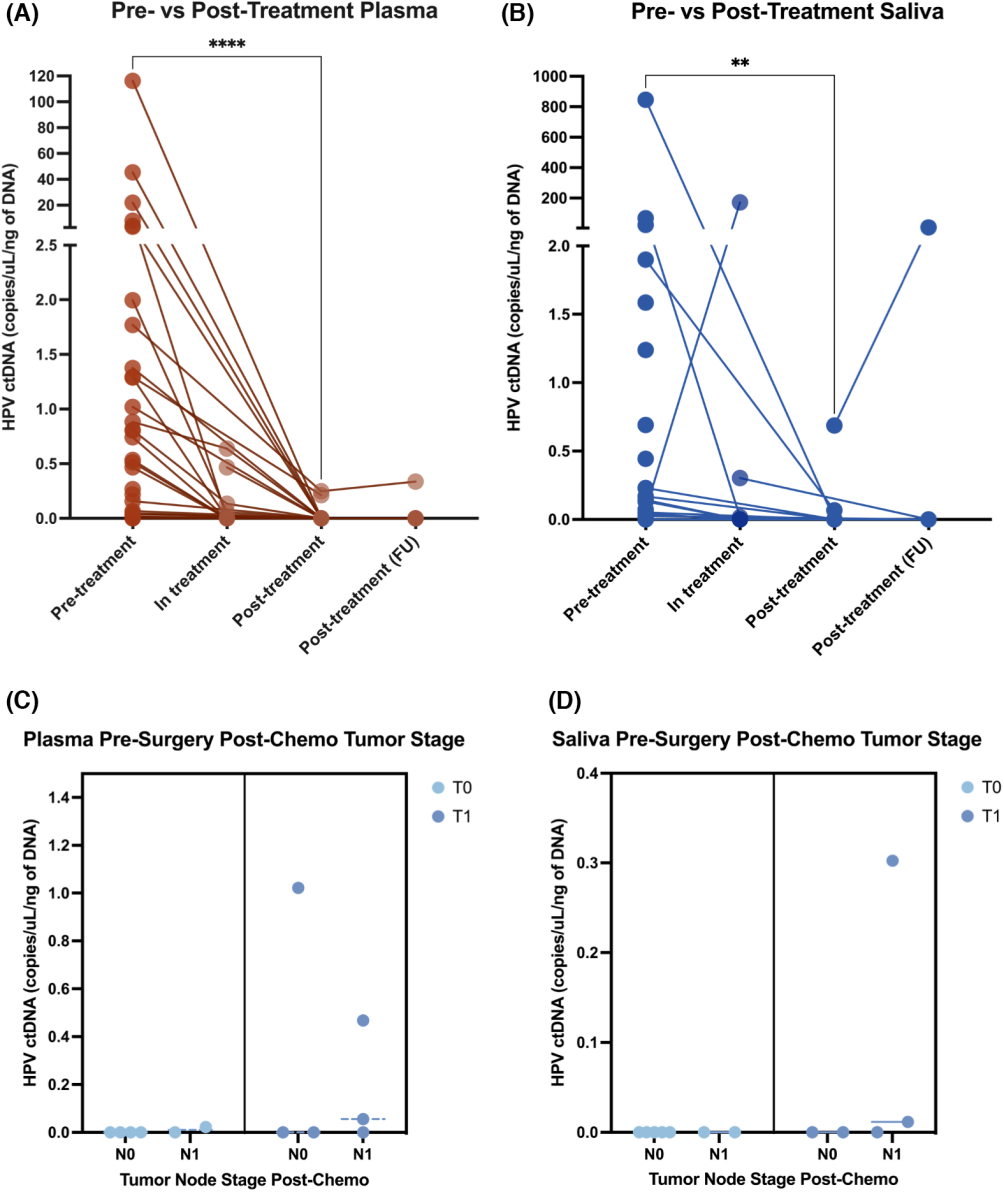
ctDNA levels decrease significantly post treatment. Pre‐treatment vs post‐treatment HPV–ctDNA levels for patients for (A) plasma and (B) saliva. Statistical comparison for pre‐ vs. post‐treatment ctDNA levels performed using a Wilcoxon matched pairs signed rank test (****p < 0.0001, **p < 0.01). Patients were classified as “in‐treatment” if they had received neoadjuvant chemotherapy prior to surgery or diagnostic positive margin surgery which received later revision. Levels of ctDNA in (C) plasma and (D) saliva in patients post‐chemotherapy based on staging from pathological analysis in surgical tissue.

Our cohort included 26 patients with matched pre‐ and post‐treatment samples. All 24 patients who were positive for ctDNA prior to treatment showed significant reductions in ctDNA levels post‐treatment as compared to baseline: 21 with complete loss of detectable ctDNA following surgery, and 3 with 84%–99% reduction (Figure 4A,B). All 21 patients with undetectable ctDNA levels after treatment had correspondingly no signs of residual disease or recurrence on 6 month follow‐up imaging. Interestingly, of the two patients with reduced but persistent ctDNA levels, both showed residual disease, suggesting that ctDNA detected remaining tumor activity.

In addition to the pre‐ and post‐treatment samples, we examined 14 patients over the course of their treatment. 5/14 patients (36%) were positive for ctDNA during their treatment, either post‐neoadjuvant chemotherapy or after debulking surgery. Patients with undetectable ctDNA after neoadjuvant chemotherapy (*n* = 9) showed relatively small tumors post‐chemo (tumor range: 0–1.2 cm, average: 0.36 cm, node range: 0‐20 mm, average: 0.44 cm, Figure 4C,D). Conversely, patients in treatment who tested positive for ctDNA (*n* = 4) demonstrated poor responses to chemotherapy or showed residual disease after surgery.

### 3.5 cfDNA fragment size was significantly affected by treatment, suggesting a complementary non‐HPV biomarker for HNC

**FIGURE 5:**
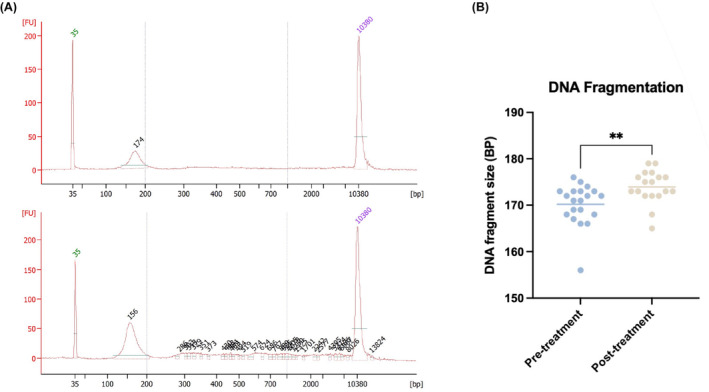
cfDNA fragment size increases post‐treatment. (A) Representative bioanalyzer results for plasma samples from 2 patients (B) Plasma DNA fragment size for matched pre‐and post‐treatment samples. Dominant fragment size chosen as fragment length with the highest concentration in plasma cfDNA using the Agilent Bioanalyzer 2100. *p* = 0.0035, paired t‐test.

While we showed that HPV sequences can be used to monitor ctDNA in HNC patients, there is currently no method to detect the presence of ctDNA without a tumor specific mutation, genomic marker or viral sequence. Recent data has suggested that fragmentation patterns of cfDNA could be a biomarker for treatment response^22^ and disease progression^23^ across various tumor types.^24^ When compared to non‐cancer specific cfDNA, tumor cfDNA has been shown to be shorter in length, likely due in part to changes in chromatin compaction and decompaction as well as differences in release mechanisms.^23^ We therefore sought to investigate the relationship between cfDNA fragment size and treatment in 20/23 matched patients for whom we had sufficient DNA. We saw an increase in dominant fragment size of plasma cfDNA after completion of treatment (*p* = 0.0035, paired *t*‐test, Figure 5), when ctDNA levels were significantly lower or undetectable. This suggests that the tumor‐specific DNA found abundantly in pre‐treatment samples may be contributing to a shorter overall fragment length, and could be a surrogate biomarker in these patients.

## 4 DISCUSSION

HPV‐related HNC cases are currently rising at an alarming rate. While this disease is associated with longer survival than its HPV‐negative counterpart, treatment often leaves patients with long‐term side effects. Furthermore, HPV+ HNC is associated with later recurrence, making non‐invasive monitoring strategies important both for detecting recurrence at the earliest stages, as well as improving overall outcomes for patients by preventing over‐treatment.

Extensive evidence from our group and others has previously shown that ctDNA from cancer patients is detectable in blood as well as in different relevant analytes such as saliva, urine, and cerebrospinal fluid.^13^, ^25^ While most work in this field focuses on identifying tumor specific mutations, recent studies have shown that HPV‐ctDNA can be detected in the blood of patients with HPV‐related HNC.^19^, ^26^ Here, we demonstrate the use of liquid biopsy from both blood and saliva to monitor treatment response and residual disease in primary and recurrent HNC patients. Our data showed a significant correlation between treatment response and ctDNA detection, suggesting an opportunity to use ctDNA as a biomarker of treatment efficacy and predictor of recurrence in this patient population.

Recently, the use of liquid biopsies to isolate ctDNA from cancer patients has given rise to new approaches to monitor tumor progression, evolution, response to treatment, and acquisition of resistance.^27^ ctDNA is short‐lived in circulation,^13^ making it a valuable tool for real‐time assessment. As cfDNA is thought to be released largely as a result of cell death,^28^ its detection can provide information on treatment response during cytotoxic treatment. Recent data suggests that early ctDNA release may reflect outcomes in cancer patients.^29^, ^30^, ^31^, ^32^, ^33^

Our first goal was to determine which analytes could be used to effectively detect HPV–ctDNA in HNC patients. We conducted a prospective, longitudinal clinical study in the blood and saliva of 60 HPV+ HNC patients. We found high concordance between saliva and blood detection of ctDNA (93%). Individually, both analytes yielded high sensitivity to detect ctDNA in patients prior to treatment. Only 3 patients were found to have no detectable HPV–ctDNA before treatment, and were found to be negative for HPV16/18/31/33/35/45. In conjunction with prior evidence^9^, ^10^ of small subsets of tumors that are p16‐positive but HPV‐negative, this implies that the lack of HPV‐ctDNA in certain patients is related to tumor HPV status as opposed to the sensitivity of the assay. This data suggests that ctDNA detection in our cohort may be higher if we account for true HPV‐association.

Subsequently, we aimed to determine whether HPV–ctDNA correlates with disease burden, similar to the effects seen using mutation‐based ctDNA tracking.^21^ HPV integration is a major event in the progression of most HPV‐related tumors, and can occur at multiple sites throughout the genome, highlighted in recent work by Symer et al.^34^ The large degree of variability seen between‐patients but not within‐patients in HPV‐ctDNA concentrations is therefore likely related to differences in tumor HPV copy number. Comparison of ctDNA levels using PCR may therefore be optimized by using an adapted baseline for each patient. Future work to determine the number of HPV integration sites underway by our group may also help improve quantification of ctDNA in a consistent way.

Along with our attempts to detect the presence of HPV‐ctDNA in patients, we aimed to examine ctDNA kinetics immediately following surgery. Recent data from O'Boyle et al. suggests that HPV‐ctDNA is cleared in the day following surgical treatment for OPC.^35^ Indeed, our data provides added evidence for the rapid clearance of ctDNA with surgery, with patients showing major decreases in ctDNA levels in the 30 min following tumor resection. Patients showed consistency in sampling immediately post‐surgery and in the following weeks, with negative ctDNA immediately post‐surgery being associated with lack of recurrence. Interestingly, one patient with a large tumor (T3N1) showed a major decrease in ctDNA immediately following surgery, but remained positive. This patient was found to be negative for HPV‐ctDNA 1 month later, implying that ctDNA not fully cleared in the first 20 min following tumor excision. In our cohort, the lack of ctDNA or decreased levels of ctDNA were maintained in follow‐up, pointing to a rapid clearance of tumor‐related DNA. Overall, our results indicate that the levels of ctDNA in patients following surgical management are an important indicator of disease status post‐surgery.

Our data additionally implies that recurrence and residual disease can be seen in ctDNA. This effect is visible both immediately after and in the months to years following treatment. Patients with positive ctDNA after treatment showed evidence of disease, either in the form of positive surgical margins or in radiological changes indicating recurrence. This data is fitting with evidence in other studies showing that ctDNA analysis can identify minimal residual disease.^27^ All but 2 patients who had no signs of recurrence or residual disease after treatment were negative for ctDNA. Over time, one of these patients showed recurrence that was not detected until 18 months after initial sampling. This data supports our hypothesis that increased ctDNA levels may be useful to detect recurrence earlier than current detection approaches. When sampling mid‐treatment, only patients with incomplete surgical management or residual tumors post‐chemotherapy showed positive ctDNA. While it is impossible to definitively say whether these findings are an indicator of future recurrence, as all patients in this category received subsequent surgical treatment, previous studies have shown that ctDNA levels after treatment are associated with recurrence risk.^20^, ^27^, ^36^, ^37^ Taken together, our data attests that HPV‐ctDNA is specifically related to residual tumor after treatment.

We also sought to investigate DNA fragmentation as a biomarker of disease. Recent data has suggested that fragment length is shorter for tumor‐specific cfDNA compared to cfDNA derived from normal cells. For this reason, shifts in cfDNA fragment length have the potential to highlight tumor burden.^22^, ^23^ Results from the fragmentation analysis in plasma samples showed significantly smaller sizes of cfDNA pre‐treatment, which fits with literature seen across numerous cancer types.^22^, ^38^ As HPV‐ctDNA levels were much higher in pre‐treatment than post‐treatment samples, this suggests that the tumor‐specific DNA may be more fragmented compared to DNA originating from non‐cancer cells. Because changes in fragment length is not disease‐specific, this data points to DNA fragment size as a complementary tool to be used along with ctDNA testing.

While both analytes (blood and saliva) were effective for the detection of HPV‐ctDNA, each presents different obstacles. In addition to intrinsic differences between the analytes, treatment for HPV‐positive HNC often includes radiotherapy, which severely limits saliva production in patients. This effect likely contributed to undetectable HPV‐ctDNA in certain patients, as an inability to generate sufficient saliva compromises the sensitivity of the assay. Conversely, while plasma displays lower variability in overall cfDNA, low plasma cfDNA levels in certain patients may have contributed to negative results in patients positive for salivary ctDNA. Our results indicate that both analytes are effective for detection of ctDNA specific to the tumor, and that their combined use enhances the sensitivity of the assay.

## 5 CONCLUSION

Our study demonstrates that HPV‐ctDNA from both plasma and saliva is an effective and highly sensitive tool for the detection of both primary and recurrent HPV‐related head and neck cancer, as well as for the detection of residual disease present after a primary treatment and to monitor tumor response over time. Together, this data provides evidence that longitudinal sampling of plasma and saliva from HPV‐related head and neck cancer patients could be a useful tool for the monitoring of disease status and the early detection of recurrence.

## AUTHOR CONTRIBUTIONS

**Sarah Tadhg Ferrier:** Conceptualization (equal); data curation (equal); formal analysis (lead); investigation (lead); methodology (lead); project administration (equal); writing – original draft (lead). **Thupten Tsering:** Investigation (supporting); methodology (supporting); validation (supporting); writing – review and editing (supporting). **Nader Sadeghi:** Data curation (equal); resources (equal); writing – review and editing (supporting). **Anthony Zeitouni:** Conceptualization (equal); funding acquisition (lead); resources (equal); writing – review and editing (supporting). **Julia V. Burnier:** Conceptualization (equal); funding acquisition (equal); methodology (supporting); project administration (lead); resources (equal); visualization (equal); writing – original draft (supporting); writing – review and editing (lead).

## FUNDING INFORMATION

This study was funded by the McGill University Health Centre Foundation. The funding body was not involved in the study design, collection, analysis, or interpretation of data.

## CONFLICT OF INTEREST STATEMENT

The authors declare that they have no competing interests.

## ETHICS APPROVAL AND CONSENT TO PARTICIPATE

Informed consent was obtained from all individual participants included in the study.

## Data Availability

The data that support the findings of this study are available from the corresponding author upon reasonable request.

## References

[1] Chaturvedi AK , Engels EA , Anderson WF , Gillison ML . Incidence trends for human papillomavirus‐related and‐unrelated oral squamous cell carcinomas in the United States. J Clin Oncol. 2008;26(4):612‐619. 18235120 10.1200/JCO.2007.14.1713

[2] Johnson DE , Burtness B , Leemans CR , Lui VWY , Bauman JE , Grandis JR . Head and neck squamous cell carcinoma. Nat Rev Dis Primers. 2020;6(1):1‐22. 33243986 10.1038/s41572-020-00224-3 PMC7944998

[3] Zeller JL . High suicide risk found for patients with head and neck cancer. JAMA. 2006;296(14):1716‐1717. 17032977 10.1001/jama.296.14.1716

[4] Song JS , Vallance P , Biron V , Jeffery CC . Epidemiological trends of head and neck cancer survivors in Alberta: towards improved understanding of the burden of disease. J Otolaryngol‐Head Neck Surg. 2020;49(1):1‐6. 32631452 10.1186/s40463-020-00443-4 PMC7339434

[5] Gillison ML , Koch WM , Capone RB , et al. Evidence for a causal association between human papillomavirus and a subset of head and neck cancers. J Natl Cancer Inst. 2000;92(9):709‐720. 10793107 10.1093/jnci/92.9.709

[6] Pfister DG , Spencer S , Adelstein D , et al. Head and neck cancers, version 2.2020, NCCN clinical practice guidelines in oncology. J Natl Compr Cancer Netw. 2020;18(7):873‐898. 10.6004/jnccn.2020.0031 32634781

[7] Chaturvedi AK , Engels EA , Pfeiffer RM , et al. Human papillomavirus and rising oropharyngeal cancer incidence in the United States. J Clin Oncol. 2011;29(32):4294‐4301. 21969503 10.1200/JCO.2011.36.4596 PMC3221528

[8] Deschuymer S , Mehanna H , Nuyts S . Toxicity reduction in the treatment of HPV positive oropharyngeal cancer: emerging combined modality approaches. Front Oncol. 2018;8:439. 30356651 10.3389/fonc.2018.00439 PMC6189290

[9] Ndiaye C , Mena M , Alemany L , et al. HPV DNA, E6/E7 mRNA, and p16INK4a detection in head and neck cancers: a systematic review and meta‐analysis. Lancet Oncol. 2014;15(12):1319‐1331. 25439690 10.1016/S1470-2045(14)70471-1

[10] Lewis JS Jr , Thorstad WL , Chernock RD , et al. p16 positive oropharyngeal squamous cell carcinoma: an entity with a favorable prognosis regardless of tumor HPV status. Am J Surg Pathol. 2010;34(8):1088‐1096. 20588174 10.1097/PAS.0b013e3181e84652 PMC3873742

[11] Mader S , Pantel K . Liquid biopsy: current status and future perspectives. Oncol Res Treat. 2017;40(7‐8):404‐408. 28693023 10.1159/000478018

[12] Kim KY , Lewis JS Jr , Chen Z . Current status of clinical testing for human papillomavirus in oropharyngeal squamous cell carcinoma. J Pathol Clin Res. 2018;4(4):213‐226. 30058293 10.1002/cjp2.111 PMC6174616

[13] Wan JC , Massie C , Garcia‐Corbacho J , et al. Liquid biopsies come of age: towards implementation of circulating tumour DNA. Nat Rev Cancer. 2017;17(4):223‐238. 28233803 10.1038/nrc.2017.7

[14] Koessler T , Paradiso V , Piscuoglio S , et al. Reliability of liquid biopsy analysis: an inter‐laboratory comparison of circulating tumor DNA extraction and sequencing with different platforms. Lab Invest. 2020;100(11):1475‐1484. 32616816 10.1038/s41374-020-0459-7

[15] Cabel L , Bonneau C , Bernard‐Tessier A , et al. HPV ctDNA detection of high‐risk HPV types during chemoradiotherapy for locally advanced cervical cancer. ESMO Open. 2021;6(3):100154. 34022731 10.1016/j.esmoop.2021.100154 PMC8164037

[16] Kaymaz Y , Oduor CI , Aydemir O , et al. Epstein‐Barr virus genomes reveal population structure and type 1 association with endemic Burkitt lymphoma. J Virol. 2020;94(17):e02007‐19. 10.1128/JVI.02007-19 PMC7431785 32581102

[17] Chan KA , Woo JK , King A , et al. Analysis of plasma Epstein–Barr virus DNA to screen for nasopharyngeal cancer. N Engl J Med. 2017;377(6):513‐522. 28792880 10.1056/NEJMoa1701717

[18] Chera BS , Kumar S , Beaty BT , et al. Rapid clearance profile of plasma circulating tumor HPV type 16 DNA during chemoradiotherapy correlates with disease control in HPV‐associated oropharyngeal cancer. Clin Cancer Res. 2019;25(15):4682‐4690. 31088830 10.1158/1078-0432.CCR-19-0211 PMC6679766

[19] Wang Y , Springer S , Mulvey CL , et al. Detection of somatic mutations and HPV in the saliva and plasma of patients with head and neck squamous cell carcinomas. Sci Transl Med. 2015;7(293):293ra104. 10.1126/scitranslmed.aaa8507 PMC4587492 26109104

[20] Gale D , Heider K , Ruiz‐Valdepenas A , et al. Residual ctDNA after treatment predicts early relapse in patients with early‐stage non‐small cell lung cancer. Ann Oncol. 2022;33(5):500‐510. 35306155 10.1016/j.annonc.2022.02.007 PMC9067454

[21] Keller L , Belloum Y , Wikman H , Pantel K . Clinical relevance of blood‐based ctDNA analysis: mutation detection and beyond. Br J Cancer. 2021;124(2):345‐358. 32968207 10.1038/s41416-020-01047-5 PMC7852556

[22] Cristiano S , Leal A , Phallen J , et al. Genome‐wide cell‐free DNA fragmentation in patients with cancer. Nature. 2019;570(7761):385‐389. 31142840 10.1038/s41586-019-1272-6 PMC6774252

[23] Van Der Pol Y , Mouliere F . Toward the early detection of cancer by decoding the epigenetic and environmental fingerprints of cell‐free DNA. Cancer Cell. 2019;36(4):350‐368. 31614115 10.1016/j.ccell.2019.09.003

[24] Udomruk S , Orrapin S , Pruksakorn D , Chaiyawat P . Size distribution of cell‐free DNA in oncology. Crit Rev Oncol Hematol. 2021;166:103455. 34464717 10.1016/j.critrevonc.2021.103455

[25] Bustamante P , Tsering T , Coblentz J , et al. Circulating tumor DNA tracking through driver mutations as a liquid biopsy‐based biomarker for uveal melanoma. J Exp Clin Cancer Res. 2021;40(1):196. doi:10.1186/s13046-021-01984-w 34134723 PMC8207750

[26] Siravegna G , O'Boyle CJ , Varmeh S , et al. Cell‐free HPV DNA provides an accurate and rapid diagnosis of HPV‐associated head and neck cancer. Clin Cancer Res. 2021;28:719‐727. 10.1158/1078-0432.CCR-21-3151 PMC8866203 34857594

[27] Pantel K , Alix‐Panabières C . Liquid biopsy and minimal residual disease—latest advances and implications for cure. Nat Rev Clin Oncol. 2019;16(7):409‐424. 30796368 10.1038/s41571-019-0187-3

[28] Jahr S , Hentze H , Englisch S , et al. DNA fragments in the blood plasma of cancer patients: quantitations and evidence for their origin from apoptotic and necrotic cells. Cancer Res. 2001;61(4):1659‐1665. 11245480

[29] Kamat AA , Bischoff FZ , Dang D , et al. Circulating cell‐free DNA: a novel biomarker for response to therapy in ovarian carcinoma. Cancer Biol Ther. 2006;5(10):1369‐1374. doi:10.4161/cbt.5.10.3240 16969071

[30] Rago C , Huso DL , Diehl F , et al. Serial assessment of human tumor burdens in mice by the analysis of circulating DNA. Cancer Res. 2007;67(19):9364‐9370. doi:10.1158/0008-5472.CAN-07-0605 17909045

[31] Cao H , Banh A , Kwok S , et al. Quantitation of human papillomavirus DNA in plasma of oropharyngeal carcinoma patients. Int J Radiat Oncol Biol Phys. 2012;82(3):e351‐e358. doi:10.1016/j.ijrobp.2011.05.061 21985946 PMC3257411

[32] Xi L , Pham TH , Payabyab EC , Sherry RM , Rosenberg SA , Raffeld M . Circulating tumor DNA as an early indicator of response to T‐cell transfer immunotherapy in metastatic melanoma. Clin Cancer Res. 2016;22(22):5480‐5486. doi:10.1158/1078-0432.CCR-16-0613 27482033 PMC7802600

[33] Husain H , Melnikova VO , Kosco K , et al. Monitoring daily dynamics of early tumor response to targeted therapy by detecting circulating tumor DNA in urine. Clin Cancer Res. 2017;23(16):4716‐4723. doi:10.1158/1078-0432.CCR-17-0454 28420725 PMC5737735

[34] Symer DE , Akagi K , Geiger HM , et al. Diverse tumorigenic consequences of human papillomavirus integration in primary oropharyngeal cancers. Genome Res. 2022;32(1):55‐70. 34903527 10.1101/gr.275911.121 PMC8744672

[35] O'Boyle CJ , Siravegna G , Varmeh S , et al. Cell‐free human papillomavirus DNA kinetics after surgery for human papillomavirus‐associated oropharyngeal cancer. Cancer. 2022;128(11):2193‐2204. doi:10.1002/cncr.34109 35139236 PMC10032347

[36] Radovich M , Jiang G , Hancock BA , et al. Association of circulating tumor DNA and circulating tumor cells after neoadjuvant chemotherapy with disease recurrence in patients with triple‐negative breast cancer: preplanned secondary analysis of the BRE12‐158 randomized clinical trial. JAMA Oncol. 2020;6(9):1410‐1415. 32644110 10.1001/jamaoncol.2020.2295 PMC7349081

[37] Tie J , Wang Y , Tomasetti C , et al. Circulating tumor DNA analysis detects minimal residual disease and predicts recurrence in patients with stage II colon cancer. Sci Transl Med. 2016;8(346):346ra92. 10.1126/scitranslmed.aaf6219 PMC5346159 27384348

[38] Underhill HR , Kitzman JO , Hellwig S , et al. Fragment length of circulating tumor DNA. PLoS Genet. 2016;12(7):e1006162. 27428049 10.1371/journal.pgen.1006162 PMC4948782

